# Hypotension and bradycardia, a serious adverse effect of piribedil, a case report and literature review

**DOI:** 10.1186/s12883-018-1230-1

**Published:** 2018-12-27

**Authors:** Piao Zhang, Yan Li, Kun Nie, Lijuan Wang, Yuhu Zhang

**Affiliations:** 1 Department of Neurology, Guangdong Neuroscience Institute, Guangdong General Hospital, Guangdong Academy of Medical Sciences, Guangzhou, 510080 Guangdong province China; 2 The Second School of Clinical Medicine, Southern Medical University, Guangzhou, 510515 Guangdong province China

**Keywords:** Piribedil, Hypotension, Bradycardia, Parkinson’s disease, Adverse drug reactions

## Abstract

**Background:**

Dopamine agonists (DAs) are efficacious for the treatment of motor and nonmotor symptoms in patients with Parkinson’s disease (PD). The treatment of PD with DAs is often complicated by adverse drug reactions (ADRs) of dopaminergic and non-dopaminergic origins. The DA piribedil is widely used in Asian, European, and Latin American countries; therefore, its ADRs are pertinent to clinicians. Here we present a rare case of hypotension and bradycardia that is significantly related to the dosage of piribedil.

**Case presentation:**

A middle-aged male, diagnosed with PD, received dopamine replacement with piribedil. When taking 50 mg piribedil daily dose, the patient didn’t feel any discomfort. Two hours after taking 100 mg piribedil he presented with serious concomitant hypotension and bradycardia with a blood pressure (BP) reading of 85/48 mmHg and a heart rate (HR) of 45 beats/min when sitting. After taking 75 mg piribedil, the patient showed the same symptoms with BP reading at 70/45 mmHg and HR of 47 beats/min in the same position. Upon replacing treatment with pramipexole 0.125 mg, 0.25 mg, and 0.375 mg three times a day, no further cardiovascular effects persisted.

**Conclusions:**

No studies have previously reported the simultaneous observation of position-unrelated hypotension and bradycardia after taking small doses of piribedil. More studies are needed to explore the effects of DAs on BP and HR, especially piribedil. Piribedil is efficacious for the treatment of PD, but it is important to weigh the potential risk of hypotension and bradycardia against the clinical benefits of this drug.

## Background

Parkinson’s disease (PD) is a neurodegenerative disease of dopamine neurons in the substantia nigra. According to a recent report, the incidence rate of PD for all age groups is between 1.5 and 22 per 100,000 person-years, placing an increasing burden on our families and society [1]. DAs used in PD treatment are a feasible supplement to levodopa, and the combined treatment has shown fewer motor complications than when levodopa is used in isolation [2]. Orthostatic hypotension is a well-known adverse effect associated with the administration of DAs at any point, and arises as an acute effect when starting the medication [3]. However, current literature on concomitant hypotensive and bradycardic effects of piribedil after oral administration has not been found.

## Case

**Fig. 1:**
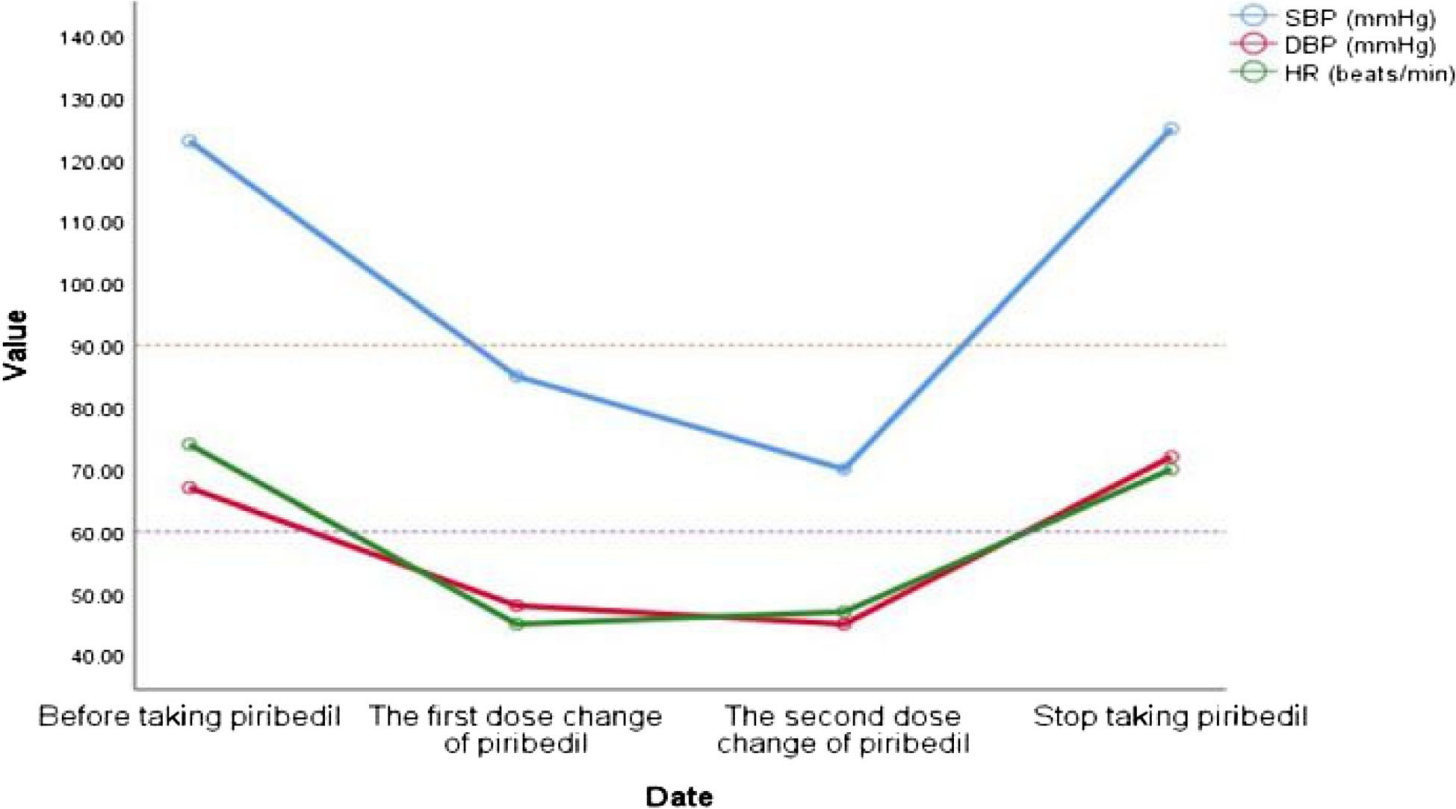
Blood pressure and heart rate fluctuation. Abbreviations: SBP = systolic blood pressure, DBP = diastolic blood pressure, HR = heart rate

A 62 year-old male had displayed bradykinesia and tremor of his right limbs for one year, during which he was able to perform limited fine movements such as dressing himself, lacing up his shoes and brushing his teeth. His tremors were aggravated by nervousness and relieved when asleep. He had had a history of hypertension and took a daily dose of 5 mg amlodipine. The patient had no history of any other chronic illnesses and was not on any other type of medication. Neither the electrocardiogram nor the Holter monitor showed any abnormalities. His baseline recumbent-upright blood pressure (BP) and heart rate (HR) were normal prior to treatment with piribedil, as shown in Table 1. He was diagnosed with PD based on the Movement Disorder Society clinical diagnostic criteria [4]. Initially, he received dopamine replacement therapy of 50 mg piribedil per day. Although there was no significant improvement in symptoms neither did he feel any discomfort. Therefore, starting the first dose change of piribedil, he added extra 50 mg to his dose. About two hours later after the first change in dose, the patient experienced symptoms of dizziness and sweating; he collapsed half an hour later. Whilst in a sitting position, the patient’s BP and HR were measured immediately. The BP reading was 85/48 mmHg and HR was 45 beats/min. His symptoms continued for the duration of the day with sitting BP fluctuating between 80–95 mmHg to 45–68 mmHg. Because his head computerized tomography examination found no abnormalities, the patient received 500 mL of 0.9% sodium chloride solution, after which his symptoms improved. Due to adverse drug reactions (ADRs), the patient was started on a second dose change of piribedil, i.e. an extra dose of 25 mg piribedil was to be taken in the afternoon in addition to the existing 50 mg taken in the morning. After two and a half hours, the patient experienced dizziness, sweating, nausea and vomiting, with a BP reading of 70/45 mmHg and a HR of 47 beats/min when sitting. His BP recovered to 105/65 mmHg and HR to 60 beats/min after elevating his lower limbs and resting for 20 mins. As a result of these incidences, the patient was switched to pramipexole to replace piribedil. After taking pramipexole 0.125 mg or 0.25 mg three times a day (tid), the symptoms of hypotension and bradycardia disappeared but a reduced amplitude of his right arm swing was still observed. Finally, after the pramipexole dose was increased to 0.375 mg tid, the patient showed marked improvements in motor symptoms. The changes in BP and HR are shown in Fig. 1.

**Table 1:** BP and HR in lying and standing position before taking and after stopping taking piribedil

		Lying	Standing for 1 min	Standing for 3 min	Standing for 5 min	Standing for 7 min
Before	BP (mmHg)	123/67	118/72	116/70	117/71	124/73
	HR (beats/min)	74	86	85	82	84
After	BP (mmHg)	125/72	122/79	125/83	129/79	126/82
	HR (beats/min)	70	89	88	86	88

## Discussion

This case demonstrated the close relationships between BP and HR with the piribedil dosage and between BP and HR with the time frame of symptom appearance, i.e. the patient exhibited symptomatic hypotension and bradycardia after approximately 2 h after administration of piribedil treatment, which is roughly the time piribedil would take to reach maximum concentration, i.e. 1 h [5]. Because of this close relationship hypotension and bradycardia were the resulting adverse effects of piribedil. After the oral administration of piribedil, the patient was initially diagnosed with anaphylactic shock [6] after observing a systolic BP decrease of greater than 30% from baseline. However, since the patient did not display any allergic reactions, he was finally diagnosed with concomitant hypotension and bradycardia; these symptoms were unlike other cases and had not been reported before.

DAs for the treatment of PD are divided into two categories; one is ergot (i.e., bromocriptine, cabergoline, lisuride and pergolide), the other is non-ergot (i.e., apomorphine, piribedil, pramipexole, ropinirole and rotigotine). All DAs possess high affinity for the D2-like family of dopamine receptors (which include the D2, D3, D4 receptors), but the ergot DAs have a higher affinity for alpha-adrenergic (alpha-1 and alpha-2) and serotonergic (5-HT1 and 5-HT2) receptors than non-ergot DAs. A review summarized a series of ADRs associated with DAs, including peripheral and central reactions [7]. The former comprises gastrointestinal reactions such as nausea and vomiting, cardiovascular reactions such as orthostatic hypotension and leg edema, while the latter includes psychotic or behavioral syndromes and sedative reactions [7]. A previous meta-analysis concluded that the recurrence of somnolence, hallucination and anxiety was much higher with non-ergot DAs, whereas the frequencies of depression, vomiting, or arterial hypotension were higher with ergot DAs [8], although it remains unclear whether this discrepancy was caused by the different receptors between ergot and non-ergot DAs. Nonetheless, it has been proven that some ADRs are associated with different receptor properties, for example, 5-HT2 receptors are potentially related to the pathogenesis of valvular heart disorders [9]; Meanwhile, stimulation of alpha-2 adrenergic receptors increases the occurrence of dyskinesias, while alpha-2 adrenergic antagonists have been shown to reduce levodopa-induced dyskinesias [10]. This is consistent with the higher incidence of dyskinesias reported for the alpha-2 adrenergic receptor agonist, pergolide [10], whilst alpha-1 adrenoreceptors are mainly associated with cardiovascular function [11]. Apomorphine hydrochloride and bromocriptine mesylate reduce BP in normal and hypertensive individuals, so they may been used in patients with hypertension [12]. There are conflicting evidences about the mechanism of hypotension in ergot DAs. In addition to decreasing the adrenal catecholamine levels, plasma aldosterone levels and renin activity associated with antihypertensive effect, the use of DAs have also evidenced improvement in renal functions, increase of inulin clearance and a decline in renal vascular resistance [12, 13]. Furthermore, ergot DAs have been proven to stimulate peripheral dopamine receptors and inhibit sympathetic nerves [13, 14]. Most of the research on DA-induced hypotension showed an accompanying increase in HR. One study suggested that the acute orthostatic hypotension might be a direct effect of dopamine receptors instead of the clinical illness or concurrent medications, which could have also resulted in lowered BP [3].

Piribedil, a representative of non-ergot DAs, is a D2/D3 dopamine receptor agonist to improve motor symptoms in early PD patients and motor fluctuations in advanced PD patients by simultaneously blocking alpha-2 adrenoreceptors and exerting minimal effects on serotoninergic, cholinergic, and histaminergic receptors, and can also improve non-motor symptoms such as vigilance, attention, memory and mood [15]. piribedil is hepatically metabolized (mainly demethylation, p-hydroxylation, and N-oxidation) and produces many metabolites, one of which is a D1 agonist [16]; Orthostatic hypotension has always been characterized as a common cardiovascular adverse effect that emerges within the first week of piribedil use, accounting for 5% of ADRs for this drug [17].

An interesting finding showed that the intravenous administration of quinpirole-dose-dependent, selective DAs could significantly lead to hypotension and bradycardia in anaesthetised normotensive rats via dopamine D2 receptors located in peripheral circulation and within the spinal cord. This finding is consistent with results seen in later studies. [18, 19]. The similar symptoms observed in this case may be a mechanism of the dopamine D2 receptors in the spinal cord. Another research, however, pointed out that intravenous administration of apomorphine produced hypotension and bradycardia through presynaptic dopamine autoreceptors [20]. In healthy subjects, the dopamine D2/D3 receptors binding in striatum relates negatively to supine resting systolic BP and HR [21]. Previous investigations have shown that D2 receptors are mainly located presynaptically on 1) sympathetic nerves where they inhibit norepinephrine release, and in 2) autonomic ganglia where they reduce ganglionic transmission once activated. The use of piribedil can directly cause hypotensive effects and a fall in HR by acting primarily on the peripheral presynaptic D2 receptor to reduce the release of the norepinephrine. Since domperidone, a D2-receptor antagonist which does not cross the blood-brain barrier, can completely block the hypotensive effects and partially block the bradycardia effects of piribedil, this may indirectly confirm that the bradycardic effect of piribedil is via central action to some extent [21, 22]. Stimulation of the dopamine receptors in the central system can result in a decrease in the sympathetic outflow.

It has been established that alpha-2 adrenoceptors are located presynaptically on the locus coeruleus neurons, which contributes centrally to autonomic functions via the release of noradrenaline [23]. Exposure to alpha-2 adrenoceptor agonists (e.g. clonidine) would mediate inhibitory effects on noradrenergic pathways, leading to increased activity of parasympathetic nervous system and reduced activity of sympathetic nervous system, thus lowering blood pressure; alpha-2 adrenoceptor antagonists, such as yohimbine, would derive the opposite effect [23]. Piribedil, with the properties of an alpha-2 adrenoreceptor antagonist, can cause autonomic-cardiovascular effects via the blockade of central and peripheral alpha-2 adrenoreceptors that inhibit the sympathetic nervous system [24]. Theoretically, piribedil may increase BP. However, in another alpha-2 receptor antagonist, atipamezole, the cardiovascular side-effects was not observed with low dose, however an increase in BP was observed with relatively higher dose [25];it remains to see with further investigation whether the same cardiovascular effect will be observed when on low doses of piribedil.

D1 receptors, primally located in vascular smooth muscle of renal and visceral beds, produce hypotensive effects via vasodilation and are accompanied by an increase in HR due to a baroreceptor reflex [26]. Piribedil may also have a role as a D1 receptor agonist due to its metabolites, however this remains to be proven [16]. In addition to its action on peripheral presynaptic D2 receptors and central system, piribedil plays a role in hypotension by stimulating the peripheral D1 receptors, which is followed by the increase in HR owing to a reflexive increase of sympathetic activity to the vasodilatory effect on the peripheral artery [13]. But the patient in our case showed an obvious decrease in HR. In summary, piribedil exerts its effect on hypotension and bradycardia possibly via D2 receptors.

Pramipexole, a non-ergot dopamine agonist with novel properties, has the highest affinity of the dopamine D2 receptor subfamily and shows preferential affinity for the D3 receptor subgroup. Apart from dopamine receptors, pramipexole also binds to alpha-2 adrenoceptors [27]. The pharmacokinetic index of pramipexole is linear and the plasma concentration increases with dosage. Pramipexole is well absorbed after administration but is hardly metabolized and is excreted unchanged in the urine. The peak plasma concentration of pramipexole occurs after oral administration for 1–3 h, with its half-life ranging from 8 to 12 h [28]. Currently, cardiovascular ADRs of pramipexole possibly include leg edema, but not orthostatic hypotension and heart failure. Orthostatic hypotension has not been associated with pramipexole, possibly because pramipexole shows higher affinity for the D3 receptors and the D1, D3, D4 dopamine receptors show no major effects on the heart; meanwhile, the D2 agonists would decrease heart rate as well as left ventricular contractility [19]. According to a meta-analysis, the use of non-ergot DAs including pramipexole in PD patients was not associated with an increased risk of incident heart failure, nor did it show an increase in the overall mortality or the risk of cardiovascular events compared with the PD patients taking monotherapy with levodopa alone [29].

In summary, piribedil can produce D1 agonist metabolites, whereas pramipexole is hardly metabolized and is delivered unchanged from urine, which may explain the lack of hypotension with pramipexole use, but not bradycardia. The lower risk of hypotension and bradycardia with pramipexole could also be understood through the perspective of receptor interactions, whereby pramipexole has higher affinity for D3 receptor.

Currently, the simultaneous appearance of hypotension and bradycardia after taking DAs is rarely observed, whether in clinical practice or research, because hypotension followed by an increase in HR has been the general observation. In addition, current studies mainly investigate orthostatic hypotension rather than position-unrelated hypotension. Based on the existing literature, the current observations of the ADRs associated with piribedil use are not a surprise. Until now it remains unknown whether the sympathetic nerve activity and renin–angiotensin–aldosterone system play an important role in hypotension and bradycardia together with piribedil. The patient in our case experienced hypotension and bradycardia associated directly with piribedil intake irrespective of standing or lying positions. This was at odds with previous reports, suggesting that piribedil may probably lead to concomitant hypotension and bradycardia via inhibition of sympathetic nerve activity and decreasing of plasma renin activity and plasma aldosterone, along with activating peripheral and central D1 dopaminergic receptor, which needs further studies to confirm. If future cases of piribedil use present similar observations, piribedil should be stopped and the situation should be remedied with fluid replacement. More research is urgently needed to explore the incidence and mechanisms of concomitant hypotension and bradycardia caused by DAs.

## Abbreviations

ADRs: Adverse drug reactions
BP: Blood pressure
DAs: Dopamine agonists
DBP: Diastolic blood pressure
HR: Heart rate
PD: Parkinson’s disease
SBP: Systolic blood pressure
